# Surgical site infections after sarcoma resections in the peripelvic region: do we need perioperative antibiotic prophylaxis?

**DOI:** 10.3389/fonc.2024.1467694

**Published:** 2024-10-18

**Authors:** Alexander Klein, Chataut Chudamani, Andreas Wieser, Sophia S. Goller, Luc M. Berclaz, Dorit Di Gioia, Boris M. Holzapfel, Hans Roland Dürr

**Affiliations:** ^1^ Orthopaedic Oncology, Department of Orthopaedics and Trauma Surgery, Ludwig-Maximilian-University (LMU) University Hospital, Ludwig-Maximilian-University (LMU) Munich, Munich, Germany; ^2^ Division of Infectious Diseases and Tropical Medicine, Ludwig-Maximilian-University (LMU) University Hospital, Ludwig-Maximilian-University (LMU) Munich, Munich, Germany; ^3^ German Centre for Infection Research (DZIF), Partner Site Munich, Munich, Germany; ^4^ Medical Microbiology and Hospital Epidemiology, Max von Pettenkofer Institute, Faculty of Medicine, LMU Munich, Munich, Germany; ^5^ Immunology, Infectious Disease and Pandemic Research (IIP), Fraunhofer Institute for Translational Medicine and Pharmacology (ITMP), Munich, Germany; ^6^ Department of Radiology, Ludwig-Maximilian-University (LMU) University Hospital, Ludwig-Maximilian-University (LMU) Munich, Munich, Germany; ^7^ Department of Internal Medicine III, Ludwig-Maximilian-University (LMU) University Hospital, Ludwig-Maximilian-University (LMU) Munich, Munich, Germany

**Keywords:** infection, sarcoma, surgery, thigh, gluteal, antibiotic, prophylaxis, perioperative

## Abstract

**Introduction:**

Surgical site infections (SSI) are one of the most common complications after extensive sarcoma resections and represent a daily challenge. SSI occur in up to 50% of cases particularly in the peripelvic area. One possible approach to reduce infection rate is perioperative antibiotic prophylaxis. The aim of this study therefore was to investigate the influence of perioperative antibiotic prophylaxis on the infection rate and the possible influence of location-specific antibiotic prophylaxis with ampicillin/sulbactam.

**Methods:**

This monocentric retrospective study included 366 patients who underwent sarcoma resections in the groin, proximal thigh, or gluteal region. All patients were operated on by 2 surgeons after neoadjuvant pretreatment if necessary. 3 groups of patients were defined. Group 1: In 60.4% of all cases, antibiotic prophylaxis was administered with cephalosporins (also clindamycin in case of penicillin allergy). Group2: In 9.8% of cases, ampicillin/sulbactam was used. Group 3: 29.8% of patients did not receive any antibiotic prophylaxis.

**Results:**

In 31.1% of treated cases, antibiotic therapy was prolonged due to extended tumor resections. Postoperative infections occurred in 23.2% (85 cases), in 77 cases within the first 90 days (on average after 20 days). The median operating time, blood loss was higher, and tumor size were significantly larger in cases with infections, compared to patients without infection. In group 1 and 2 with perioperative single-shot prophylaxis, infection occurred in 24.1% of cases, compared to 13.5% of cases without prophylaxis (group 3) (p= 0.032). In the patients with prolonged antibiotic therapy, infection occurred in 31.6% of cases, compared to 16.3% of cases without prolongation (p< 0.001). In the group 2, infection occurred in 19.4% of cases compared to 24.9% of cases in the group 1 (p= 0.479). In the multivariate analysis, surgery time longer 80 min, blood substitution, neoadjuvant radio- and chemotherapy proved to be a risk factor for SSI.

**Discussion:**

Region adapted perioperative antibiotic prophylaxis may reduce the risk of infection after extended sarcoma resection in the peripelvic area. However, the particular bacterial spectrum of this anatomic region should be taken into account when deciding which antibiotics to use.

## 1 Introduction

Soft tissue sarcomas (STS) are rare diseases and occur with an incidence of around 50 per million inhabitants per year (1). Almost 50% of extraabdominal STS are located at the thigh or gluteal region (2). Due to the complex anatomy of this region and often large tumor extensions, the resection of these sarcomas is complex and causes long operation times. The complex dissection during surgery and proximity to the large blood vessels also frequently cause major loss of blood during or after surgery. The loss of soft tissues regularly causes large wound cavities in which hematomas and seromas may develop. In addition, neoadjuvant radiotherapy and/or chemotherapy increase the risk of wound healing disorders in many cases (3, 4). All these factors lead to a significantly increased risk of infection after resection of sarcomas in the peripelvic region (5–7). This is aggravated by the particular spectrum of bacteria in this region, which differs significantly from other regions (8–10). In previous studies (also in our own patient collective (7)), high proportions of Gram-negative and anaerobic pathogens were found (5, 6). It is also known that moist skin (as seen in the groin) has significantly more pathogenic germs (10) which might be brought into the wound mechanically during surgery.

Major efforts are and have been taken to reduce the infection rate in various studies. In addition to general antiseptic measures, this includes antibiotic prophylaxis. However, in recent literature some of the recommendations in musculoskeletal surgery are based on little evidence and there are hardly any studies on this topic. The recommendations from the 2000s recommend a differentiated approach to antibiotic prophylaxis (11). Published in 2013, the Surgical Infection Society did not recommend antibiotic prophylaxis for aseptic procedures without the implantation of prostheses at all (12). Due to the general high infection rate and the personal experience of the authors, antibiotic prophylaxis nevertheless seems to be advisable for complex resections of sarcomas in the peripelvic area. Depending on the complexity of surgery, antibiotics may also be administered on a prolonged basis. However, these are clinically based individual decisions for which evidence is currently lacking, and only sparse preliminary work in the recent literature can be found.

The aim of this study therefore was to evaluate the risk factors for the development of postoperative infections after resections of sarcomas in the groin, proximal thigh, and gluteal region. Furthermore, the effectiveness of perioperative antibiotic prophylaxis should be evaluated also in cases with prolonged antibiotic administration. In addition, we evaluated the effectiveness of an adaption of antibiotics for prophylaxis, considering the particular spectrum of bacteria in this region found by previous evaluations of our cohort.

## 2 Patients and methods

In a historical patient collective of a specialized sarcoma center, patients were selected and their data retrospectively evaluated who underwent STS resections of the proximal thigh (till 15 cm distal to the groin), groin or gluteal region (peripelvic region) at our center between 2003 and 2020. Patients with the following characteristics were included:

- Resection of a STS in the peripelvic region.
- Tumor resection in our hospital.
- No superficial sarcomas.
- Primary wound closure without application of vacuum-assisted closure systems (VAC).
- Clinical follow-up time of at least 2 weeks.
- Occurrence of infection in the first 90 days after surgery.
- Availability of follow-up data for the first 90 days after surgery.

Almost all tumors were diagnosed in advance by biopsy (incisional or core-needle biopsy). The only exceptions were atypical lipomatous tumors (ALT), which were only diagnosed radiologically. Treatment decision was made in an interdisciplinary tumor board. Many tumors were neoadjuvantly treated in accordance with the ESMO (European Society for Medical Oncology) guidelines (13). Depending on the entity, grading, TNM classification of the tumor, and the individual age, patients received chemotherapy and/or radiotherapy. Patients were then operated on exclusively by 2 experienced surgeons. Depending on the interdisciplinary decision, adjuvant therapy was performed if necessary. While follow-up, all patients were seen at our center at least in the first 3 months. In the event of a postoperative infection after discharge from hospital, all patients presented again at our center.

The decision for preoperative single shot antibiotic prophylaxis was based on clinical assessment and the complexity of the resection. Due to the lack of evidence and recommendations for pre-/perioperative antibiotic prophylaxis, the decision for or against antibiotic administration was made historically based on clinical experience of surgeons from the 1990s and 2000s. No antibiotic was administered for tumors with a size <10 cm and epifascial location. If antibiotic prophylaxis was desired, it was administered 30 minutes before the incision. If the operation lasted longer than 3 hours and/or blood loss exceeded 2 litres, antibiotics were administered repeatedly. Extended postoperative prophylaxis for 5-7 days was ordered, if the duration of surgery exceeded 2 hours or if intraoperative blood loss exceeded 1000 ml. Primarily, the 2nd generation cephalosporin (cefuroxime 1.5 g) was administered. In cases of known penicillin allergy, a lincosamide antibiotic substance (clindamycin 600 mg) was applied as an alternative. The interim evaluation of the bacterial spectrum in wound infections 2018 has shown a high proportion of Gram-negative and anaerobic bacteria, many of which were associated with intestinal flora (coagulase-negative staphylococci (CoNS) in 31.5%, Enterococcus spp. in 13.3% and Escherichia coli 7.7%). 30.8% microbial species were Gram-negative bacteria, 25.9% were anaerobic species (7). Adapted to this finding, the prophylaxis was changed to ampicillin/sulbactam 2 g/1 g in consecutive patients.

In the event of a deep wound infection requiring revision, a surgical revision was performed. Postoperative deep wound infection was defined following the guidelines of the Center for Disease Control and Prevention (CDC) (14). The wound was explored, at least two microbiological swabs were taken under sterile conditions, and the wound was debrided and either closed primarily or left for secondary wound healing with VAC therapy. Antibiotics were administered according to the antibiograms of the isolated pathogens.

The following parameters were evaluated: gender, age of the patient, co-morbidities (body mass index (obesity), Diabetes mellitus, active nicotine consumption, arteriosclerosis), duration of surgery, intraoperative blood loss, substitution of erythrocyte concentrates, neoadjuvant and/or adjuvant therapy, tumor entity, tumor size, application and type of perioperative antibiotic prophylaxis, occurrence of infection, type of pathogens, change of pathogens during the treatment.

For data analysis we used descriptive statistic methods (frequency, median value, spreading, standard deviation and Kaplan-Meier curve). Significance analysis was performed using the Log-Rank, Chi-Square, or the t-test, defining a 95% confidence interval. Univariate (Cox proportional hazards regression) and multivariate analysis were used to evaluate the influence of group characteristics on the infection rate. The level of significance was set at less than 0.05. The data analysis software used was IBM^®^ SPSS^®^ Statistics 29. This study was approved by the institutional ethics committee (Ethics Committee of the Medical Faculty; ID 17-89). Written consent was obtained from all surviving patients. For non-surviving patients, data were irreversibly anonymized as recommended by the ethics committee. For children and adolescents informed consent from their parents/guardians was obtained.

## 3 Results

### 3.1 Cohort characteristics

A total of 393 patients were evaluated. 27 patients had to be excluded from the evaluation because the data was incomplete or the minimum observation period of 3 months was not reached. In total, 366 patients could be evaluated. Of those, 204 patients (55.7%) were male. The median age of all patients was 61.3 years (range between 9 and 93 years). The median time of surgery was 79.8 min (range between 15 and 620 min). The median intraoperative blood loss was 474.9 ml (range between 40 and 8300 ml). The median maximum length of the resection specimen was 16.1 cm (range between 4 and 65 cm). Other group characteristics are shown in **Table 1**.

**Table 1:** Characteristics of patient cohort and treatment details.

	Total Number/Total Cohort	Infection Free Group	SSI Group	Power (95% CI)
**Number of Patients**	366	289 (79%)	77 (21%)	
Sex				
**Female**	162	128 (44.3%)	34 (44.2%)	0.983
**Male**	204	161 (55.7%)	43 (55.8%)	
**Age (Years)**	61.3	62.5	61	0.542
**Body Mass Index**	26.1	25.7	27.1	**0.022**
Diabetes mellitus				
**Yes**	40	30 (10.7%)	10 (11.8%)	0.778
**No**	326	251 (89.3%)	75 (88.2%)	
Arteriosclerosis				
**Yes**	31	20 (7.1%)	11 (12.9%)	0.091
**No**	335	261 (92.9%)	74 (87.1%)	
Active smoker				
**Yes**	39	28 (10%)	11 (12.9%)	0.436
**No**	327	253 (90%)	74 (87.1%)	
**Surgery time (min)**	79.8	73.9	101.1	**< 0.001**
**Tumor size (cm)**	16.1	15.5	18.5	**0.009**
**Blood lost (ml)**	474.9	397	767	**0.005**
Blood substitution				
**Yes**	49	28 (9.7%)	21 (27.3%)	**< 0.001**
**No**	317	261 (90.3%)	56 (72.7%)	
Neoadjuvant Chemotherapy				
**Yes**	126	104 (36%)	22 (28.6%)	0.250
**No**	240	185 (64%)	55 (71.4%)	
Adjuvant Chemotherapy				
**Yes**	109	86 (29.8%)	23 (29.9%)	0.906
**No**	257	203 (70.2%)	54 (70.1%)	
Neoadjuvant Radiotherapy				
**Yes**	103	77 (26.6%)	30 (39%)	**0.026**
**No**	263	212 (73.4%)	47 (61%)	
Adjuvant Radiotherapy				
**Yes**	123	98 (33.9%)	25 (32.5%)	0.871
**No**	243	191 (66.1%)	52 (67.5%)	
Perioperative Prophylaxis				
**Yes**	257	195 (67.5%)	62 (80.5%)	**0.032**
**No**	109	94 (32.5%)	15 (19.5%)	
Postoperative Prophylaxis				
**Yes**	114	78 (27%)	36 (46.8%)	**< 0.001**
**No**	252	211 (73%)	41 (53.2%)	

SSI, surgical site infection; CI, confidence interval. Bold value: statistically relevant differences.

**Figure 1:**
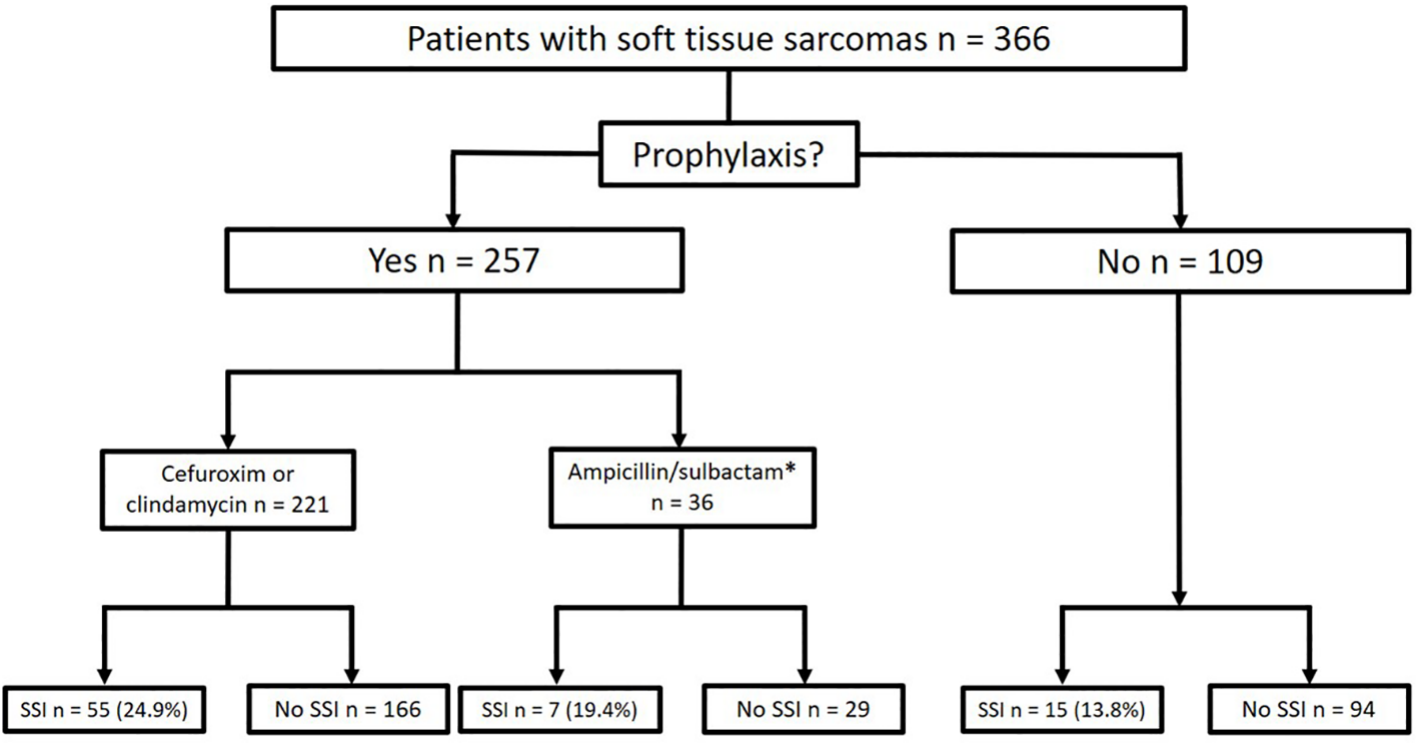
Frequencies of antibiotic prophylaxis and rates of postoperative infection (SSI, Surgical Site Infection; *after the interim evaluation of the pathogen spectrum in the patient cohort, antibiotic prophylaxis was switched to ampicillin/sulbactam).

Antibiotic prophylaxis was administered as a single shot perioperatively in 257 cases (70.2% of all patients), 29.8% of patients did not receive any prophylaxis because of low risk constellation. Cefuroxime or clindamycin was administered in 221 cases (56.3% of all patients), and ampicillin/sulbactam was given in 36 cases (9.8% of all patients, **Figure 1**). In 114 cases (31.1% of all cases or 51.6% of cases with antibiotic prophylaxis), antibiotic prophylaxis was continued postoperatively due to the complexity of surgery (**Table 1**).

### 3.2 Rate of SSI

SSI occurred in 85 cases (23.2% of all operations). Eight cases of infection occurred later than 90 days after the index surgery and were excluded from further analysis. They were considered as probable superinfections of the pre-existing seroma due to hematogenous spread. These cases were excluded for the evaluation of our study aim. In 77 cases (21% of all operations), the median time to diagnosis of infection and surgical revision was 20 days (range between 3 and 90 days). The mean time of surgery, blood loss, and tumor size differed significantly (p<0.05) between the group with and without infections (**Table 1**).

### 3.3 Risk factors for development of SSI

62 infections (out of 257, 24.1%) occurred in the group with perioperative antibiotic prophylaxis. In the group without antibiotic prophylaxis 15 infections occurred in 109 cases (13.8%; p=0.032). However, both groups differed highly significantly in baseline values as tumor size (p=0.007) or blood loss and duration of surgery (both p<0.001, **Table 2**).

**Table 2:** Subgroup characteristics and it’s comparison regarding tumor size (cm, centimeter); blood loss (ml, milliliter) and surgery time (min, minutes).

	Tumor Size (cm)	Blood Loss (ml)	Surgery Time (min)
Perioperative Prophylaxis			
Yes	16,78 **p=0.007**	576,27 **p<0.001**	88,11 **p<0.001**
No	14,74	229,36	61,35
Prolonged prophylaxis			
Yes	18,78 **p<0.001**	839,91 **p<0.001**	110,11 **p<0.001**
No	15,00	309,71	66,79
Prophylaxis with Cefuroxime or clindamycin vs. Ampicillin/Sulbactam	16,58 p=0.186	548,31 p=0.108	85,68 p=0.323
	18,10	786,29	100,14

Bold value: statistically relevant differences.

In the group with single shot antibiotics, 41 infections occurred in 252 cases (16.3%; p<0.001) compared to 36 (31.6%) infections in 114 cases with prolonged antibiotic prophylaxis (**Figure 1**). Also here, the group characteristics (tumor size, blood loss and duration of surgery differed highly significantly (p<0.001; **Table 2**).

**Figure 2:**
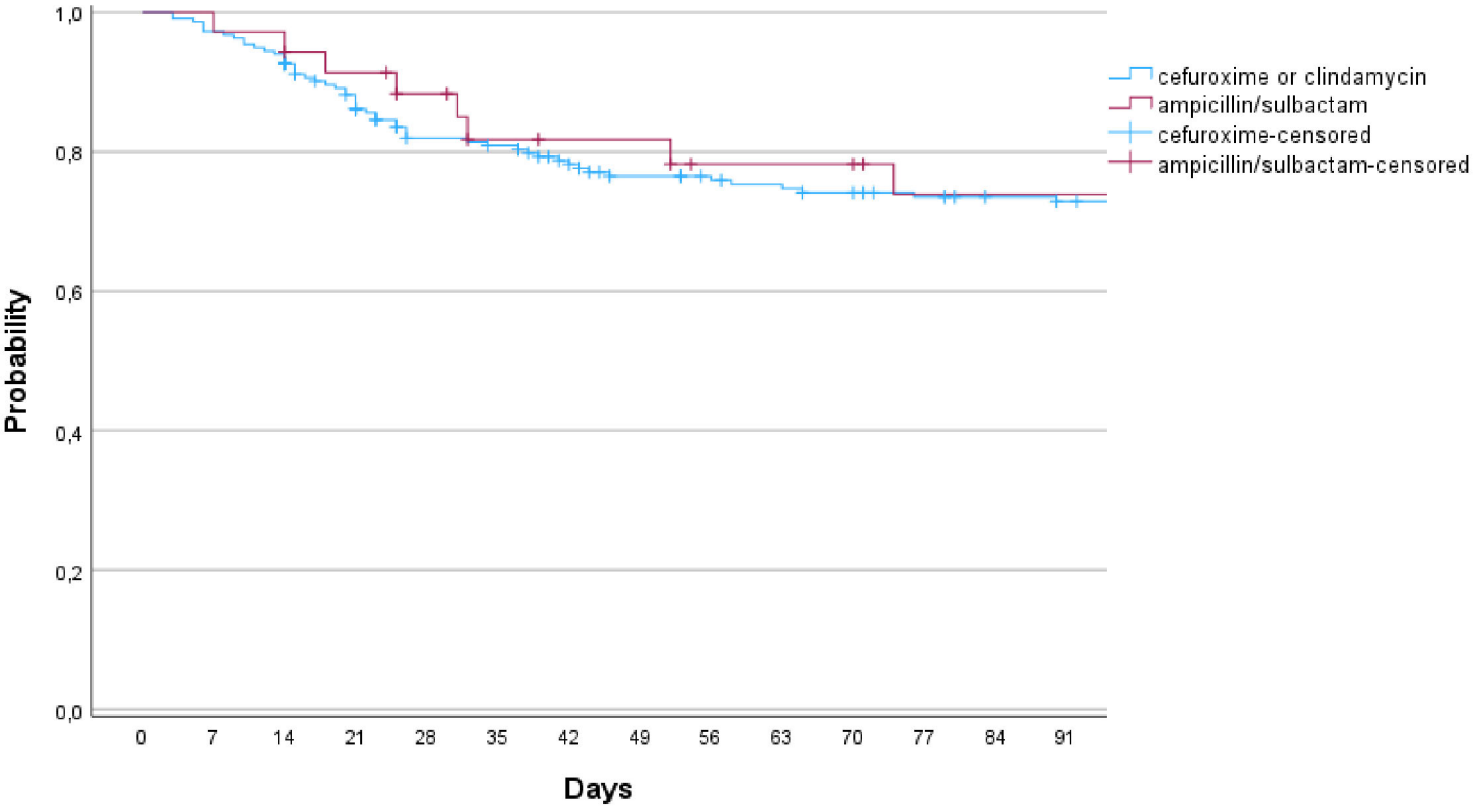
Infection free survival in comparison of the group of cefuroxime (or clindamycin) and ampicillin/sulbactam (p=0.775; 95% CI).

In comparison of the groups with cefuroxime (including clindamycin) and ampicillin/sulbactam, 55 infections occurred in 221 cases (24.9%) in the cefuroxime (and clindamycin) group, whereas 7 infections in 36 cases (19.4%; p=0.479) occurred in the ampicillin/sulbactam group (**Figure 2**, n.s.). The groups were comparable in terms of tumor size (p=0.186), blood loss (p=0.108), and time of surgery (p=0.323; **Table 2**).

In univariate analysis, the blood loss ≥ 800 ml, substitution of erythrocyte concentrates, surgery duration longer 80 min. and neoadjuvant radiotherapy were found to be significant risk factors for the development of infection (**Table 3**). In multivariate analysis, the surgery duration longer 80 min, substitution of erythrocyte concentrates and neoadjuvant radiotherapy were confirmed as a risk factor. In addition, the implementation of neoadjuvant chemotherapy significantly worsened the risk for SSI (**Table 3**).

**Table 3:** Univariate and multivariate analysis of the risk factors, influencing the development of SSI.

Factor	Univariate Analysis	Multivariate Analysis
**Obesity (BMI ≥ 30 kg/m^2^)**	0.065	0.192
**Diabetes mellitus**	0.778	0.351
**Arteriosclerosis**	0.091	0.102
**Active smoker**	0.436	0.208
**Surgery time ≥ 80 min.**	**<0.001**	**0.006**
**Blood loss ≥ 500 ml.**	**0.004**	0.476
**Tumor size > 15 cm**	0.146	0.915
**Blood substitution**	**<0.001**	**0.008**
**Neoadjuvant chemotherapy**	0.251	**0.023**
**Neoadjuvant radiotherapy**	**0.013**	**0.029**
**Adjuvant chemotherapy**	0.386	0.317
**Adjuvant radiotherapy**	0.772	0.412

BMI, body mass index; power 95% CI. Bold value: statistically relevant differences.

## 4 Discussion

The resection of STS in the peripelvic region maybe a challenge for the surgeon. Complex anatomy and a large extent of the tumor often causes long surgery durations with high blood loss. Those are known risk factors for the development of SSI. This is frequently aggravated by neoadjuvant or adjuvant chemotherapy and radiotherapy. Precisely, these risk factors are confirmed in a large meta-analysis by *Slump* et al. (4): The evaluation of 21 studies on STS resections at all sites showed an overall complication rate of 30.2% with a surgical revision rate of 13.4%. The risk factors identified included large tumors, neoadjuvant radiotherapy, and high intraoperative blood loss. The infection rate in patient cohorts with mixed localizations is around 15-18% (15, 16), while in pelvic bone sarcomas or soft tissue sarcomas in the peripelvic area the infection rate is about 20-25% (5, 6). In concordance with these findings, the SSI rate was 21% (7).

In current guidelines a uniform recommendation from different societies is seen: the American Surgical Infection Society recommends no antibiotic prophylaxis for aseptic orthopaedic operations without using implants (12). The recommendation of the German Paul-Ehrlich-Society is identical (17). However, risk factors for an increased risk of SSI in these procedures are also identified: Neoadjuvant radiation, surgery duration over 2 h, long anaesthesia time and high blood loss with blood transfusion. If these facts are taken into account, there is a tendency to advocate additional methods to reduce the infection rate. General aseptic measures have long been established in clinical practice: Aseptic washing, avoiding skin injuries, and using iodized films (12). However, there is no published evidence or recommendation for perioperative antibiotic prophylaxis in this cohort.

The initial hypothesis of this study was that antibiotic prophylaxis reduces the infection rate after sarcoma resection in the peripelvic area. This could not be confirmed by our evaluation. The SSI rate in all subgroups was higher with antibiotic prophylaxis compared to no prophylaxis at all. This result is certainly due to the pre-selection of patients into the respective groups (bias). The surgeons selected patients with more complex tumor resections into the groups with antibiotic prophylaxis and, if necessary, prolonged antibiotic administration based on clinical experience and assessment of the risk of infection.

However, significant differences between the groups were evident: Blood loss was higher in the group with antibiotic prophylaxis, the frequency of application of erythrocyte concentrates was higher, the duration of surgery was longer, and tumor size was more extensive. This confirms a profound selection bias regarding the administration of antibiotic prophylaxis. Other authors assume that the probability of postoperative infection in this group would even be higher without antibiotic prophylaxis. Infection rates of up to 50% have been described for these localizations (18–20). It is difficult to interpret the results in the group with prolonged antibiotic application: The infection rate was significantly higher in this group compared to a single shot (32 vs. 16%). *Dadras* et al. were also unable to achieve a reduction in the risk of infection through prolonged antibiotic administration (15), the Paris group showed the same results (20). Probably the only prospective and randomized study on the effectiveness of antibiotic prophylaxis in musculoskeletal oncology is the PARITY trial. Here, in a prospective double blinded and randomized trial, no benefit of prolonged antibiotic prophylaxis (1 day vs. 3 days) after bone sarcoma resection and implantation of tumor prostheses of the lower extremity could be shown (21). A study with a similar design would probably be useful to clarify this issue more precisely.

The analysis of the bacterial spectrum in postoperative infections in the peripelvic area showed a significantly higher proportion of Gram-negative and anaerobic pathogens, strongly associated with intestinal flora (7). Few studies investigated postoperative infection in sarcoma resections in this area, all showed similar results (5, 6). Because of this, antibiotic prophylaxis with cephalosporins appears inadequate. We, therefore, changed the prophylaxis to ampicillin/sulbactam. The comparison of these 2 groups from our patient cohort shows a trend in the reduction of infection rate (19% versus 24%), but the effect was not significant. In a retrospective evaluation of *Ramsey* et al. (22) the addition of metronidazole as an antibiotic prophylaxis to cover anaerobes reduced significantly the complication rate after sarcoma resections (27% vs. 17%; p = 0.049) underlying our own findings. In view of these results and the close relationship of the gluteal and inguinal region to the anus and, in women, to the vagina, a relation between the infectious microorganisms and the corresponding local bacterial flora can be assumed. Thus, the adaptation of antibiotic prophylaxis seems unavoidable. However, two large analyses of antibiotic prophylaxis in colorectal surgery and hysterectomy cannot clearly prove these connections (23, 24). A conclusive assessment is not possible in view of these results, but a trend is recognizable.

Resection of vessels with prosthesis reconstruction and resection of abdominal/retroperitoneal organs played no role in our cohort in terms of numbers (four in the our patient collective). In such cases, a higher SSI rate would have been expected.

The retrospective design of this study and the lack of randomization only allow limited conclusions to be drawn about the effectiveness of the prophylaxis due to the existing bias. Our results can only be interpreted as a trend. As far as our study design allows, we identified the following factors in the risk stratification for postoperative infection: Neoadjuvant radiotherapy and chemotherapy, duration of surgery, and substitution of erythrocyte concentrates as surrogate parameters for higher blood loss. The analysis of complications after surgery in nearly 1.000 STS at different locations (15) identified almost the same risk factors; further studies also confirm these results (3, 16).

There is no doubt that further studies with larger numbers of patients using the multicenter prospective approach are necessary in order to adequately answer the important question of the effectiveness of perioperative antibiotic prophylaxis.

## 5 Conclusion

The evaluation of treatment outcomes after resection of peripelvic STS in our patients could not demonstrate a benefit of perioperative antibiotic prophylaxis or prolonged antibiotic therapy in lowering the postoperative infection risk. We attribute that to a selection bias of antibiotic prophylaxis at all. However, in line with the published literature, we identified subgroups with an increased risk of infection: Obesety, longer duration of surgery, higher blood loss with the need for blood substitution, larger tumor extension, and the application of neoadjuvant radiotherapy. In our opinion, patients with a combination of these risk factors should receive antibiotic prophylaxis that ideally considers the increased Gram-negative and anaerobic bacterial spectrum in this body region. With that at least a trend for a lesser rate of SSI was seen.

## Data Availability

The original contributions presented in the study are included in the article/supplementary material. Further inquiries can be directed to the corresponding author.
